# Hippocampal Subregion Function and Its Clinical Correlations in Childhood Autism Spectrum Disorders

**DOI:** 10.1002/aur.70124

**Published:** 2025-10-02

**Authors:** Hui‐Xian Li, De‐Sheng Xuan, Ronghao Mu, Chi Qin, Xin Zhao

**Affiliations:** ^1^ Department of Clinical Research and Translational Medicine The Third Affiliated Hospital of Zhengzhou University Zhengzhou China; ^2^ Department of Radiology The Third Affiliated Hospital of Zhengzhou University Zhengzhou China; ^3^ Department of Child Developmental Behavior The Third Affiliated Hospital of Zhengzhou University Zhengzhou China

**Keywords:** autism spectrum disorder, child, clinical symptoms, functional connectivity, hippocampus

## Abstract

The hippocampus plays a crucial role in memory and social processing, both of which are impaired in autism spectrum disorder (ASD). Investigating the functional activity of hippocampal subregions can provide valuable insights into their involvement in ASD‐related social and behavioral symptoms. This study analyzed hippocampal resting‐state functional connectivity (rsFC) in 507 male child participants from the ABIDE dataset (225 ASD, 282 typical controls) and its relation to clinical features. The hippocampus was subdivided into rostral and caudal subregions, and rsFC patterns were compared between groups. Significant group differences were observed in the left caudal, right rostral, and right caudal hippocampus, with enhanced connectivity to widespread cortical and subcortical regions, including visual, motor, parietal, and cerebellar networks. Machine learning using hippocampal rsFC achieved modest classification performance. Clinically, rsFC correlated with core ASD symptoms: social awareness was associated with right caudal connectivity to fusiform and temporal regions, while restricted and repetitive behaviors were linked to distinct rostral–caudal patterns involving frontal, motor, and cerebellar areas. Age of onset showed positive correlations with rsFC across all subregions, with rostral hippocampus engaging socioemotional and motor control networks and caudal hippocampus connecting more strongly to visual and sensorimotor integration regions. These findings demonstrate subregional specificity of hippocampal connectivity in ASD, suggesting distinct anterior–posterior contributions to symptom expression and developmental timing.

**Summary**

- Significant group differences were identified in the left caudal, right rostral, and right caudal hippocampus, showing enhanced rsFC with visual, motor, parietal, and cerebellar networks.
- Subregional connectivity patterns were associated with core autism spectrum disorder (ASD) symptoms: right caudal connectivity correlates with social awareness, while rostral–caudal patterns link to restricted and repetitive behaviors.
- Age of onset was positively correlated with rsFC across all hippocampal subregions, with rostral regions engaging socioemotional and motor control networks, and caudal regions more strongly connected to visual and sensorimotor integration areas.

## 1 Introduction

The hippocampus is a key brain region involved in memory, spatial navigation, and emotional regulation (Amaral and Lavenex 2007). Autism spectrum disorder (ASD) is a neurodevelopmental disorder characterized by social deficits, restricted interests, and repetitive behaviors (Hirota and King 2023). While extensive research has explored the neurobiological correlates of ASD, the findings remain inconsistent, and studies specifically focusing on hippocampal function in ASD are relatively limited (Banker et al. 2021).

With growing recognition of the hippocampus's role in social cognition, there has been increasing attention on hippocampal changes in individuals with ASD. Some studies have revealed differences in the hippocampus at structural, functional, and molecular levels (Nicolson et al. 2006; Rexrode et al. 2024), emphasizing its potential role in autism‐related cognitive and social deficits. However, structural studies on hippocampal volume have shown inconsistent results, partly due to the heterogeneity of overall brain volume (Reinhardt et al. 2020). Notably, the hippocampus can be anatomically divided into anterior and posterior regions, each with distinct functional roles (Ayhan et al. 2021; Nadel et al. 2013). Research showed that an individual's memory ability is more closely linked to the relative size of these subdivisions than to the overall hippocampal volume (Poppenk and Moscovitch 2011). Ignoring this differentiation may lead to inconsistent findings and limit our understanding of the hippocampus's role in ASD. Recent work further demonstrates that the functional specialization of anterior and posterior hippocampal connectivity differs in individuals with ASD, highlighting that distinct hippocampal subregions may contribute differently to cognitive and social processes (Kember et al. 2024).

To date, only a few studies have directly investigated hippocampal function in individuals with ASD (Banker et al. 2021), but existing research indicates significant differences in its activity. For instance, task‐based fMRI studies have found that the functional connectivity (FC) between the hippocampus and caudate nucleus in individuals with ASD may reflect deficits in cognitive map construction (Solomon et al. 2015). Notably, studies on structural connectivity and molecular levels have also revealed different connections between the hippocampus and other brain regions. For example, changes in white matter tracts have been observed between the hippocampus and the middle fusiform gyrus (Conturo et al. 2008). Moreover, chemical metabolites in the amygdala‐hippocampus region are altered in ASD (Endo et al. 2007). Structural connectivity provides a foundation for function, and these findings suggest that the FC between the hippocampus and other brain regions may also be different. Some studies have found that during rest, individuals with ASD show enhanced FC between the hippocampus and the default mode network (Eilam‐Stock et al. 2014), whereas during episodic memory retrieval, hippocampal FC across the brain is generally reduced (Cooper et al. 2017). Emerging theories view the hippocampus as an organizer of information, especially through cognitive mapping. It supports not only memory and spatial reasoning but also the tracking of social relationships (Banker et al. 2021). These perspectives suggest that hippocampal differences may be linked to certain aspects of the ASD phenotype. Therefore, the hippocampus may be a key brain region in ASD research, and its function should be studied within the broader context of neural networks.

Building on the research background and existing gaps, this study aims to systematically investigate the specific characteristics of hippocampal function in ASD and to explore the potential mechanisms linking hippocampal dysfunction to core symptoms of ASD. We subdivided the hippocampus into rostral (anterior) and caudal (posterior) regions, examining their roles as key brain areas and investigating their FC with other brain regions at the whole‐brain level in individuals with ASD. Based on the above background, this study proposes the following hypotheses: First, resting‐state functional connectivity (rsFC) of the hippocampus differs between individuals with ASD and typically developing controls, with distinct patterns observed along the anterior–posterior axis. Specifically, alterations in anterior hippocampal connectivity are expected to primarily involve regions associated with social cognition, emotion, and higher‐order cognitive functions, such as the prefrontal cortex and basal ganglia, whereas differences in posterior hippocampal connectivity are anticipated to predominantly involve regions related to visual, motor, and sensory integration, including occipital and cerebellar areas. Furthermore, we hypothesize that hippocampal functional connectivity is significantly associated with ASD symptoms, including social deficits and repetitive behaviors, and that different hippocampal subregions may contribute differentially to specific symptom dimensions. By adopting this fine‐grained analysis approach, we hope to provide a more precise understanding of hippocampal dysfunction in ASD.

## 2 Methods

### 2.1 Participants

All data in this study were obtained from the publicly available ABIDE/ABIDEII dataset (https://fcon_1000.projects.nitrc.org/indi/abide/) (Di Martino et al. 2017, 2014). Imaging data included male ASD patients aged 6–12 years with IQ scores above 70. The typical control (TC) group was matched to the ASD group. Given the significant gender effect in ASD, only male participants were included in this study. In addition, we performed quality control on the imaging data, including checks for data coverage completeness, spatial registration quality, and head motion (mean frame‐wise displacement Jenkinson < 0.2 mm). To address site‐related issues, we only retained sites with at least 10 participants in each group. A total of 507 participants were included in the final analysis (ASD: 225, TC: 282). Participant details for each site are presented in Table S1.

### 2.2 MRI Data Preprocessing

We used DPABI toolbox (http://rfmri.org/DPABI) to perform data preprocessing (Yan et al. 2016). The first 10 time points of the participants' resting‐state fMRI time series were removed to ensure signal stabilization. Slice timing correction was applied by shifting the signal measured in each slice to the midpoint of each repetition time. Head motion correction was performed using a six‐parameter rigid‐body linear transformation using a two‐step procedure. After realignment, individual T_1_‐weighted images were co‐registered to the mean functional image using a six degrees of freedom linear transformation and segmented into gray matter, white matter (WM), and cerebrospinal fluid (CSF). Nuisance covariates, including the linear trend, head motion (Friston 24 parameters), WM signal, and CSF signal, were regressed out from the functional signal. The functional images in individual space were then transformed to MNI space using the DARTEL tool (Ashburner 2007). Finally, the normalized functional images underwent band‐pass temporal filtering (0.01–0.1 Hz) and spatial smoothing with a 6 mm FWHM kernel.

### 2.3 rsFC Analysis Based on Hippocampal Subregions

The present study employed hippocampal anatomical parcellations defined by the Human Brainnetome Atlas (www.brainnetome.org), which delineates the hippocampus into rostral and caudal portions, with two subregions in each hemisphere, yielding four subregions in total. We extracted the average time series from the four subregions and calculated the resting‐state functional connectivity strength (Pearson correlation) with all other brain voxels, followed by Fisher's *z*‐transformation. Subsequently, we applied ComBat harmonization (https://github.com/Jfortin1/ComBatHarmonization) to remove site effects. Finally, independent‐sample *t*‐tests were performed while controlling for age, IQ, and head motion as covariates. In addition, Gaussian random field (GRF) theory was applied for multiple comparison correction.

### 2.4 Machine Learning

This study employed machine learning to compare the discriminatory performance of resting‐state functional connectivity features from different hippocampal subregions in classifying individuals with ASD and TC. Functional connectivity features were first extracted from each participant based on masks highlighting subregions with significant group differences. Three classification models were then constructed: linear support vector machine, logistic regression, and random forest (RF). Model evaluation was conducted using five‐fold cross‐validation, with data standardized within each fold. Analyses were performed both for each significant subregion individually and for the combined features of all significant subregions.

### 2.5 Clinical Scales Assessment

Autism Diagnostic Observation Schedule (ADOS): The ADOS is a standardized, semi‐structured assessment tool designed to evaluate communication, social interaction, play, and restricted and repetitive behaviors associated with ASD (Lord et al. 2000). The ADOS is widely regarded as a gold standard for diagnosing ASD and is used in both clinical and research settings. The Autism Diagnostic Interview‐Revised (ADI‐R) is a semi‐structured diagnostic interview administered by clinicians, primarily with parents or primary caregivers, to assess developmental history and current behavior in children and adults, aiding in the diagnosis of ASD. It is one of the internationally recognized gold‐standard diagnostic instruments and is often used in conjunction with ADOS (Lefort‐Besnard et al. 2020). Social Responsiveness Scale (SRS): The SRS is a standardized assessment tool designed to measure the severity of social impairments related to ASD (Sturm et al. 2017). It evaluates social awareness, social cognition, social communication, social motivation, and restricted interests or repetitive behaviors across different age groups. Repetitive Behavior Scale‐Revised (RBS‐R): The RBS‐R is a standardized questionnaire designed to assess the severity and subtypes of repetitive behaviors commonly observed in ASD (Lam and Aman 2007). It evaluates six domains of repetitive behaviors: stereotyped behaviors, self‐injurious behaviors, compulsive behaviors, ritualistic behaviors, sameness behaviors, and restricted interests.

The four scales were administered to 144 (ADOS), 92 (ADI‐R), 121 (SRS), and 58 (RBS‐R) ASD participants, respectively. For the stereotyped behaviors and restricted interests (STEREO_RESTRICT) subdimension of the ADOS, 13 participants had missing data, leaving a final analysis sample of 131 participants. The ADI‐R questionnaire had missing data in the SOCIAL and ONSET subdimensions for 1 and 11 participants, respectively, resulting in final sample sizes of 91 and 81 for analyses of these two subdimensions.

### 2.6 Hippocampal rsFC and ASD Clinical Features

We then examined the relationship between rsFC strength of different hippocampal subregions and ASD symptom severity to link brain measures with behavioral measures. This analysis used ComBat‐harmonized FC data and performed partial correlation analyses at the whole‐brain level, controlling for age, IQ, and head motion, with GRF correction applied for multiple comparisons. We used four scales for analysis: ADOS, ADI‐R, SRS, and RBS‐R. ADOS provides a comprehensive assessment of ASD, while SRS and RBS‐R focus on its two core symptoms: social impairments and repetitive behaviors, allowing for a more detailed differentiation. Compared with the ADOS, the ADI‐R offers notable diagnostic advantages. Moreover, it captures developmental history information, allowing further investigation of the relevance of imaging measures for early symptom identification.

## 3 Results

### 3.1 Altered Hippocampal rsFC in ASD

**FIGURE 1:**
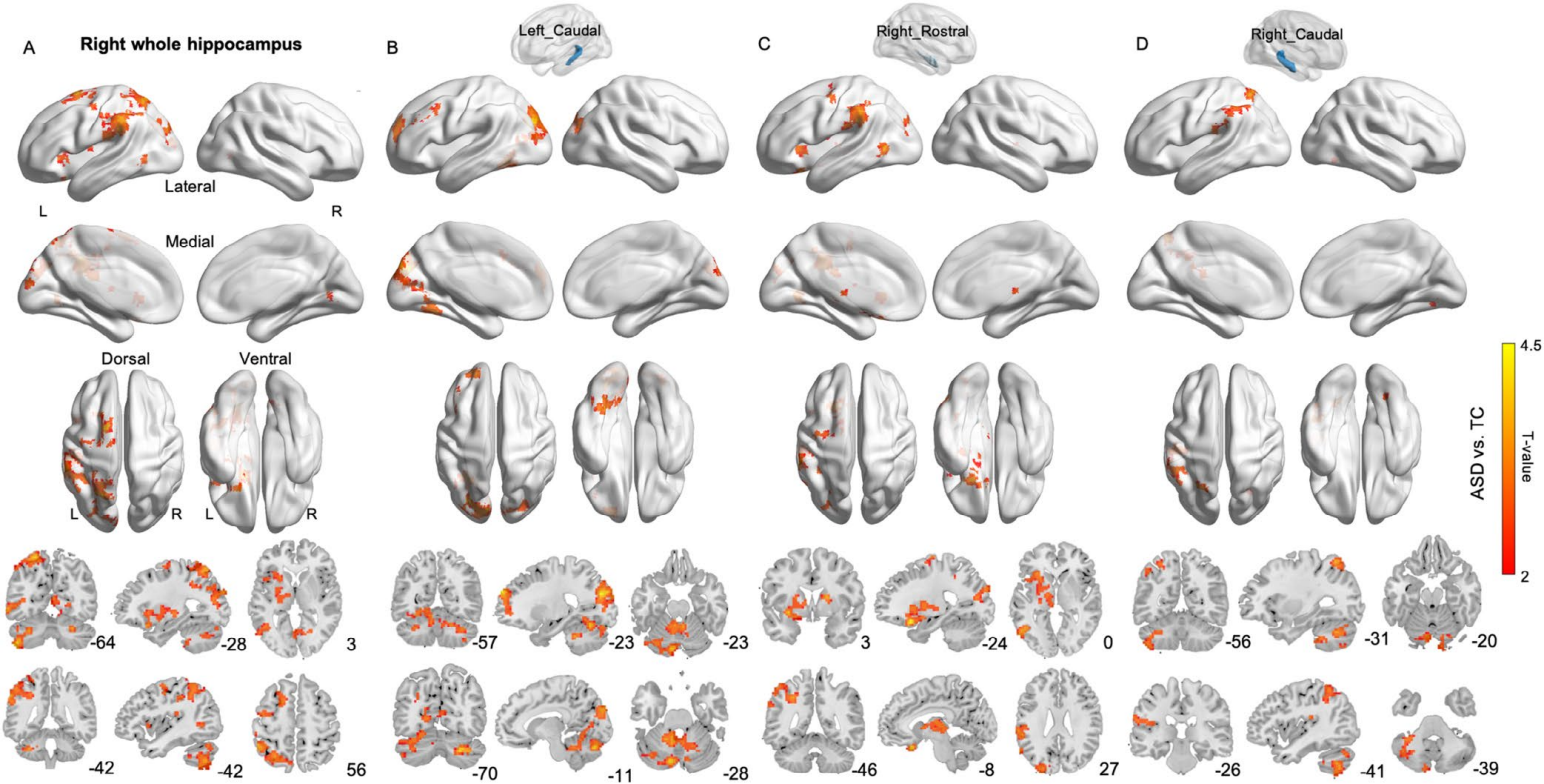
The intergroup differences in rsFC between the hippocampal subregions and other brain voxels in the ASD and TC groups. The independent‐sample *t*‐tests were performed while controlling for age, IQ, and head motion as covariates. GRF was applied for multiple comparison correction.

We first analyzed the hippocampus as a whole, without dividing it into anterior and posterior portions. No significant group differences were observed in the left hippocampus, whereas the right hippocampus showed significant differences, primarily involving the left motor and sensory cortices, parietal and occipital visual areas, as well as parts of the cerebellum and basal ganglia (Figure 1A, Table S2).

Subsequently, the hippocampus was subdivided along the longitudinal axis into rostral (anterior) and caudal (posterior) portions. Compared with TC, individuals with ASD exhibited altered resting‐state functional connectivity between hippocampal subregions and other brain voxels, predominantly in the left caudal hippocampus (1817 voxels), right rostral hippocampus (1358 voxels), and right caudal hippocampus (934 voxels), whereas no significant group differences were found in the left rostral hippocampus.

Specifically, the left caudal hippocampus exhibited significantly increased connectivity with visual and occipital regions, followed by parietal and frontal areas, and also engaged widespread cerebellar regions (Figure 1B, Table S2). The right rostral hippocampus showed markedly enhanced connectivity with the basal ganglia and thalamus, as well as the motor cortex, frontal lobe, and parts of the parietal–occipital cortex (Figure 1C, Table S2). The right posterior hippocampus demonstrated significantly increased connectivity primarily with the parietal lobe and cerebellum, and to a lesser extent with motor and visual regions (Figure 1D, Table S2).

### 3.2 Machine Learning Classification of Hippocampal rsFC in ASD and TC

Using a random forest classifier based on resting‐state functional connectivity of hippocampal subregions, we evaluated its ability to discriminate ASD from TC (Table 1). Classification using single subregions yielded modest performance, with accuracy ranging from 0.536 to 0.560 and AUC values between 0.511 and 0.547. The left caudal hippocampus showed the highest AUC among individual regions (0.547), while the right caudal hippocampus achieved the highest F1‐score (0.642). Sensitivity was generally higher than specificity across all models, indicating that hippocampal connectivity features more reliably identified ASD cases than controls. Combining multiple hippocampal subregions (Fused) slightly improved overall sensitivity (0.745) and F1‐score (0.657), but AUC remained low (0.540), suggesting limited discriminative power. These results indicate that hippocampal resting‐state connectivity carries partial information useful for ASD classification, with the caudal subregions, particularly the right caudal hippocampus. The performance of the linear support vector machine and logistic regression classifiers was inferior to that of the random forest classifier; however, the comparisons across different subregions yielded consistent results, with the right caudal hippocampus showing the best performance (Table S3).

**TABLE 1:** Classifier results based on significant between‐group rsFC differences.

Region	Accuracy	Sensitivity	Specificity	Precision	F1‐score	AUC
Left caudal	0.550	0.720	0.338	0.577	0.640	0.547
Right rostral	0.536	0.684	0.351	0.569	0.622	0.511
Right caudal	0.560	0.709	0.373	0.587	0.642	0.525
Fused	0.568	0.745	0.347	0.588	0.657	0.540

*Note*: Fused: Significant differences from the three hippocampal subregions were combined for classifier training. These results represent the model evaluation using a Random Forest classifier with five‐fold cross‐validation.

### 3.3 Correlations Between Hippocampal rsFC and Clinical ASD Features

We further investigated the correlation between rsFC strength of different hippocampal subregions and corresponding clinical assessments of ASD symptoms.

**FIGURE 2:**
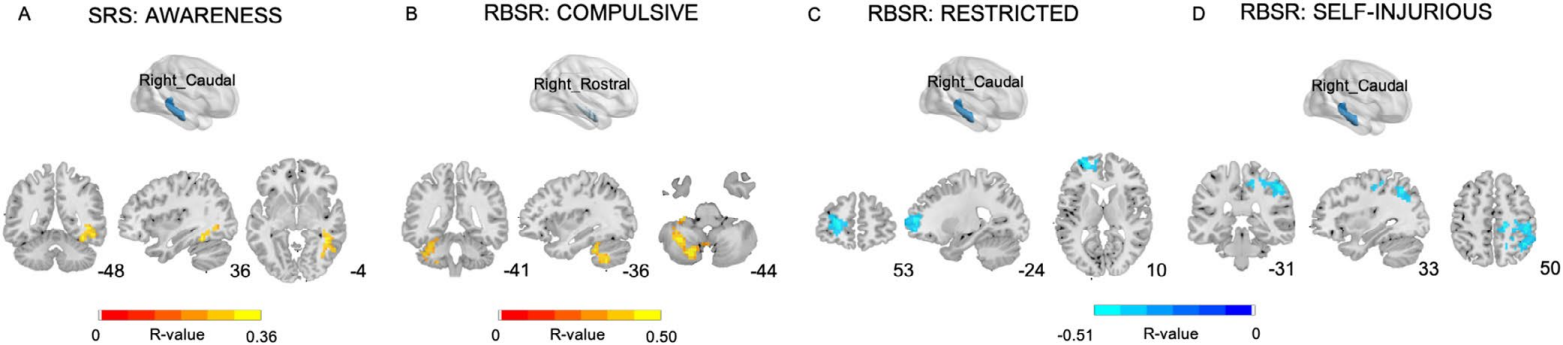
Hippocampal rsFC in ASD and its correlation with clinical features. ComBat‐harmonized FC data were used in this analysis, and partial correlation analyses were performed at the whole‐brain level, with age, IQ, and head motion controlled as covariates. GRF correction was applied for multiple comparisons. SRS: Social Responsiveness Scale; RBS‐R: Repetitive Behavior Scale–Revised. AWARENESS: Social awareness subscale of the SRS. COMPULSIVE: Compulsive behaviors subscale of the RBS‐R. RESTRICTED: Ritualistic behaviors subscale of the RBS‐R. SELF‐INJURIOUS: Self‐injurious behaviors subscale of the RBS‐R.

The SRS social awareness subscale was positively associated with rsFC between the right caudal hippocampus and the right fusiform gyrus, middle temporal gyrus, and inferior temporal gyrus (Figure 2A, Table S4). For RBS‐R subscales, compulsive behaviors were positively correlated with the connectivity of the right rostral hippocampus with left cerebellar regions and the left fusiform gyrus (Figure 2B, Table S4). Restricted behaviors showed significant negative correlations with connectivity between the right caudal hippocampus and left superior, middle, and medial superior frontal gyrus (Figure 2C, Table S4). Furthermore, self‐injurious behaviors were negatively associated with the connectivity of the right caudal hippocampus with right precentral and postcentral gyrus, paracentral lobule, supplementary motor area, superior and inferior parietal lobules, supramarginal and angular gyrus, and precuneus (Figure 2D, Table S4).

### 3.4 Correlations Between Hippocampal rsFC and Age of Onset in ASD

**FIGURE 3:**
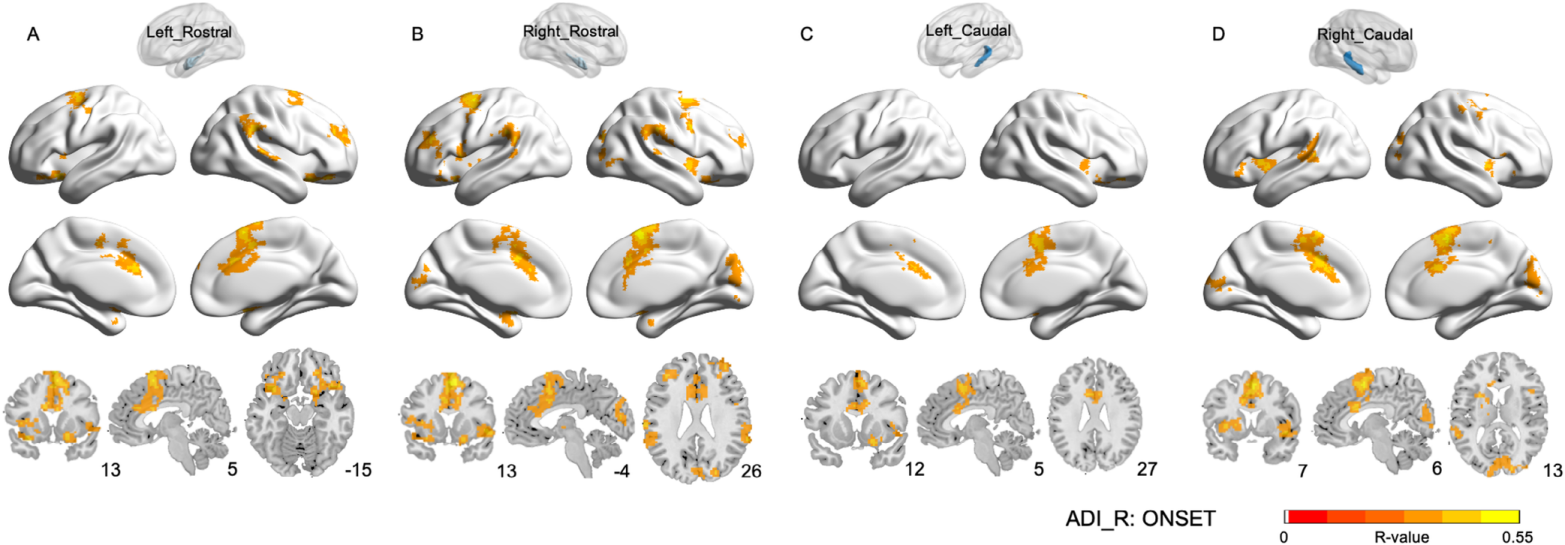
Hippocampal rsFC in ASD and its correlation with age of onset in ASD. ComBat‐harmonized FC data were used in this analysis, and partial correlation analyses were performed at the whole‐brain level, with age, IQ, and head motion controlled as covariates. GRF correction was applied for multiple comparisons. ADI‐R: Autism Diagnostic Interview‐Revised. ONSET: Abnormality of development evident at or before 36 months' total scores.

We used the ADI‐R to investigate the relationships between resting‐state functional connectivity of different hippocampal subregions and age of onset in ASD. Significant positive correlations were observed across all four hippocampal subregions (Figure 3).

In the rostral hippocampus, rsFC of the left rostral subregion with frontal areas (superior, middle, and inferior frontal gyrus, as well as the orbitofrontal cortex), motor‐related regions (precentral gyrus, supplementary motor area), insula, middle and anterior cingulate cortex, rectus gyrus, amygdala, and putamen was positively correlated with age of onset (Figure 3A, Table S5). Additional positive correlations were observed with temporal regions (superior temporal gyrus and temporal pole) and parietal areas (inferior parietal lobule, supramarginal gyrus, and angular gyrus). The right rostral hippocampus displayed a similar pattern but with broader involvement of occipital visual regions (calcarine sulcus, cuneus, superior/middle occipital gyrus), subcortical structures (putamen, pallidum, thalamus), and temporal regions (superior temporal gyrus and temporal pole) (Figure 3B, Table S5). Collectively, both rostral subregions were engaged in networks supporting cognitive regulation, motor control, emotional processing, and social cognition, with the right rostral hippocampus showing a stronger role in visual and cross‐network integration.

For the caudal hippocampus, the left subregion showed positive correlations with the right inferior frontal gyrus (opercular and orbital parts), bilateral supplementary motor area, olfactory cortex, rectus gyrus, posterior orbitofrontal cortex, insula, middle/anterior cingulate cortex, and putamen (Figure 3C, Table S5). In contrast, the right posterior hippocampus exhibited more extensive positive correlations with frontal regions (superior, middle, and inferior frontal gyrus), motor areas (precentral gyrus, supplementary motor area, paracentral lobule), insula, middle/anterior cingulate cortex, as well as occipital regions (calcarine sulcus, cuneus, superior/middle occipital gyrus), parietal areas (inferior parietal lobule, supramarginal gyrus, paracentral lobule), and temporal regions (superior/middle temporal gyrus and temporal pole) (Figure 3D, Table S5). Compared with the left caudal hippocampus, the right subregion demonstrated a broader connectivity profile, particularly with visual and sensorimotor integration regions.

## 4 Discussion

This study focused on hippocampal function in young children with ASD, dividing the hippocampus into subregions and examining differences in FC between these subregions and other brain areas. Furthermore, we analyzed the relationship between hippocampal FC and ASD clinical symptoms as well as age of onset. We found that children with ASD exhibited different FC in hippocampal subregions compared to TCs, and these connectivity patterns were significantly associated with clinical symptom assessments. Furthermore, FC of different hippocampal subregions was significantly associated with the age of onset in ASD.

We found significant differences in resting‐state functional connectivity of hippocampal subregions between individuals with ASD and TC. Specifically, the right rostral hippocampus exhibited increased connectivity with the basal ganglia (caudate, putamen, and globus pallidus), thalamus, and prefrontal cortex in the ASD group, suggesting its potential involvement in the regulation of prefrontal and deep cortical networks, which may contribute to deficits in cognitive control, action planning, and social behavior. The left caudal hippocampus in ASD primarily showed altered connectivity with visual–occipital regions and the cerebellum, while the right caudal hippocampus was mainly connected to the parietal cortex and cerebellum, indicating that the caudal hippocampus is preferentially involved in visuospatial processing and motor coordination networks. These alterations may underlie common ASD impairments in spatial perception, motor coordination, and sensory integration. Overall, anterior hippocampal connectivity is biased toward prefrontal and deep cortical networks, whereas posterior hippocampal connectivity predominantly involves posterior cortical and cerebellar networks. This anterior–posterior functional heterogeneity may reflect selective influences of hippocampal subregions on cognitive, perceptual, and motor functions in ASD. These findings are also supported by previous studies. For example, Ayhan et al. conducted single‐nucleus RNA sequencing on anterior and posterior hippocampal tissues resected from epilepsy patients, identifying significant cellular and molecular diversity along the hippocampal anterior–posterior axis (Ayhan et al. 2021). In comparison with the anterior hippocampus, the posterior hippocampus plays a crucial role in spatial memory, spatial cognition, and mental health (Nadel et al. 2013; Poppenk and Moscovitch 2011; Schmitt et al. 2009). Kember et al. reported reduced connectivity between the posterior hippocampus and posterior parietal cortex in individuals with ASD, suggesting a potential impact of the posterior hippocampus on spatial memory (Kember et al. 2024). Our findings support the functional differentiation of hippocampal subregions and may help explain the specific cognitive impairments observed in children with ASD.

We employed machine learning approaches, based on the significant differences between the two groups, to evaluate the potential clinical utility of the above findings and to compare the contributions of different hippocampal subregions. Classification based on rsFC of hippocampal subregions showing significant group differences achieved moderate accuracy, indicating that these features capture meaningful distinctions between the ASD and control groups. We further trained models using resting‐state functional connectivity between hippocampal subregions and the whole‐brain voxels (Table S6). Compared with whole‐brain features, classification performance based on significant hippocampal subregions was comparable. Sensitivity was slightly higher for the fused whole‐brain features (0.766), with a modest increase in F1‐score (0.660), but specificity was slightly lower (0.302–0.307), and AUC showed minimal change (0.514–0.555). These results suggest that rsFC of hippocampal subregions showing significant group differences already captures the key information for classification, and adding whole‐brain features only marginally improves ASD identification, providing limited gain in overall accuracy. Overall, the rsFC of these significant hippocampal subregions represents a valuable source of information for distinguishing ASD from controls. However, hippocampal features alone were insufficient for high‐accuracy classification. Future studies may benefit from integrating connectivity measures across multiple regions or multimodal imaging features to enhance predictive performance.

This study found that hippocampal rsFC in individuals with ASD was closely associated with the severity of clinical symptoms, further supporting the hippocampus' critical role in ASD. We found that social awareness was positively correlated with the functional connectivity strength between the right caudal hippocampus and the right fusiform gyrus, middle temporal gyrus, and inferior temporal gyrus. This finding has important neurobiological implications. The right temporal regions, particularly the fusiform gyrus, have been widely recognized as critical nodes for face perception, facial expression recognition, and social information processing (Weiner and Grill‐Spector 2012). The hippocampus, as a hub for episodic memory and contextual binding, has its posterior subregion more strongly implicated in memory retrieval and spatial navigation (Poppenk et al. 2013). Thus, stronger functional coupling between these regions may reflect a more efficient process of encoding and retrieving social information, allowing individuals to more effectively process, integrate, and recall social cues, thereby exhibiting enhanced social awareness (Banker et al. 2021). With respect to restricted and repetitive behaviors, our findings reveal a more fine‐grained dissociation. Compulsive behaviors were positively correlated with connectivity between the right rostral hippocampus and the cerebellum/fusiform gyrus, suggesting that this behavioral pattern may be associated with hyperactivity in neural circuits involved in habit formation, procedural memory, and sensorimotor integration (D'Mello and Stoodley 2015). In contrast, both restricted behaviors and self‐injurious behaviors were strongly negatively correlated with connectivity between the right caudal hippocampus and the prefrontal as well as sensorimotor cortices. The prefrontal cortex is responsible for higher‐order executive functions, behavioral flexibility, and impulse control (Friedman and Robbins 2022; Reynaga et al. 2020), while the parietal sensorimotor network supports somatosensory perception and motor planning (Ariani et al. 2022; Gale et al. 2021). Reduced functional coupling of the caudal hippocampus with these regulatory regions may reflect a neural mechanism characterized by insufficient contextual updating of behavior, diminished behavioral flexibility, and impaired regulation of the perception of one's own actions, thereby increasing the risk of stereotyped behaviors and self‐injury.

By examining the relationships between hippocampal subregional resting‐state functional connectivity and age of onset in individuals with ASD, this study highlights the pivotal role of the hippocampus in neurodevelopment and its potential clinical relevance. We found that rsFC strength across all hippocampal subregions was positively associated with age of onset, suggesting that more effective hippocampal connectivity with widespread brain networks may delay the emergence of ASD symptoms. This finding is consistent with the neural compensation hypothesis (Reuter‐Lorenz and Cappell 2008), which posits that the hippocampus, through its coordinated activity with cortical regions, may partially offset the impact of early neurodevelopmental abnormalities. From a developmental neurobiological perspective, the hippocampus, as a core structure of the limbic system, exhibits functional specialization along its anterior–posterior axis. The anterior hippocampus is more engaged in affective regulation and motivational processes, while the posterior hippocampus is preferentially involved in memory integration and spatial processing. In our study, the left rostral hippocampus showed positive associations with later onset age through connectivity with prefrontal regions, motor‐related regions, as well as the insula, anterior cingulate cortex, and amygdala. These areas subserve cognitive control, emotion regulation, and sensorimotor integration, suggesting that the left rostral hippocampus may contribute to delaying symptom onset by interacting with higher‐order cognitive and emotional networks (Bathelt and Geurts 2021; Colic et al. 2025). Notably, the right rostral hippocampus displayed a broader connectivity profile, involving not only these regions but also occipital visual areas and subcortical structures. This pattern implies a specific role of the right hippocampus in integrating visual information with emotional memory, and its stronger cross‐network integration may underlie a later onset of ASD symptoms. Such hemispheric asymmetry could reflect distinct developmental inputs, with the right hippocampus more specialized in visuospatial processing, which may be particularly critical for environmental adaptation and social learning in ASD. For the posterior hippocampus, we observed notable hemispheric differences. The left caudal hippocampus was primarily connected with the right inferior frontal gyrus, bilateral supplementary motor areas, olfactory cortex, and insula regions implicated in olfactory memory, motor planning, and impulse control. By contrast, the right caudal hippocampus exhibited a broader pattern of positive associations, encompassing prefrontal, sensorimotor, visual, parietal, and temporal cortices. This widespread cross‐modal integration (visual–motor–cognitive) may provide greater adaptive learning capacity (Uddin 2021), thereby delaying the manifestation of ASD core symptoms.

In summary, the anterior hippocampus demonstrated onset‐related connectivity mainly within fronto‐limbic and social‐cognitive networks, emphasizing its role in affective and higher‐order cognitive regulation. The posterior hippocampus, in contrast, showed onset‐related connectivity with a wider fronto‐parieto‐occipito‐temporal network, underscoring its contribution to visuospatial and sensorimotor integration. These anterior–posterior and hemispheric differences highlight the selective influence of hippocampal subregions on neurodevelopmental trajectories in ASD and point toward the potential value of tailoring interventions to hippocampal subregional characteristics for personalized clinical strategies. Future studies could benefit from longitudinal designs to characterize the developmental trajectories of hippocampal connectivity in children with ASD, thereby revealing the dynamic changes across different subregions during neurodevelopment. Moreover, integrating multi‐omics approaches, such as single‐cell sequencing and spatial transcriptomics, may help elucidate the molecular and cell‐type–specific mechanisms underlying these alterations, ultimately informing the development of temporally precise and target‐specific interventions.

It is noteworthy that the present study was hypothesis‐driven and focused on ROI‐based analyses. To further address the concern that ROI‐based analyses might overemphasize hippocampal contributions, we also conducted exploratory whole‐brain region‐to‐region connectivity analyses (see Supporting Information: whole‐brain analysis). These analyses revealed widespread group differences, including alterations in hippocampal connectivity (Figures S1 and S2), but none of the findings survived FDR correction. Such null results are not unexpected, as whole‐brain approaches often face a heavy multiple‐comparison burden, which can obscure subtle yet meaningful effects. Importantly, this observation suggests that functional connectivity alterations in ASD may not reflect a diffuse, global disturbance across the entire brain, but rather represent relatively specific changes concentrated within the hippocampus and its interactions with a limited set of networks. Both ROI‐based and whole‐brain analyses are meaningful, but our study was hypothesis‐driven, aiming not at unbiased exploratory search but at a focused, detailed examination of functionally relevant hippocampal subregions. We believe this approach is critical for understanding the complex role of the hippocampus in ASD. However, it should be noted that ROI‐based analyses inherently involve targeted selection of regions, and thus their interpretability depends on the a priori hypotheses guiding the choice of ROIs. In this context, combining insights from both exploratory whole‐brain analyses (for transparency) and focused ROI analyses (for sensitivity and mechanistic insight) provides a balanced framework for investigating region‐specific alterations in ASD.

## 5 Conclusions

This study provides converging evidence for altered hippocampal resting‐state functional connectivity in individuals with ASD, with distinct patterns along the anterior–posterior axis. While no group differences emerged in the left rostral hippocampus, widespread alterations were observed in the left caudal, right rostral, and right caudal subregions, implicating visual, parietal, motor, cerebellar, and subcortical networks. Machine learning analyses further highlighted that hippocampal connectivity features, particularly from caudal subregions, carry partial but limited discriminative power for distinguishing ASD from typical controls. Importantly, rsFC alterations were linked to both symptom severity and age of onset. Social awareness and repetitive behavior dimensions showed distinct associations with hippocampal–cortical and hippocampal–cerebellar coupling, while earlier onset was related to reduced engagement of rostral hippocampal networks supporting higher‐order cognitive, motor, and socio‐emotional functions. Together, these findings underscore the functional heterogeneity of hippocampal subregions in ASD and suggest that their divergent connectivity patterns may contribute to both the clinical phenotype and developmental trajectory of the disorder. It should be noted that this study included only male participants, which may limit the generalizability of these findings to females with ASD.

## Conflicts of Interest

The authors declare no conflicts of interest.

## Supporting information


**Data S1:** Supporting Information.

## Data Availability

The data that support the findings of this study are available in the ABIDE dataset (https://fcon_1000.projects.nitrc.org/indi/abide/).
