# Supplementary material for: Hippocampal Subregion Function and Its Clinical Correlations in Childhood Autism Spectrum Disorders
Source: Autism Res. 2025 Oct 2;18(11):2231–9. doi: 10.1002/aur.70124 (PMC12661258; doi:10.1002/aur.70124)
Supplement: Supplementary file 1 — Data S1: Supporting Information. [file AUR-18-2231-s001.docx]

**Table S1** | Included sites and corresponding participant counts.

| Site Name | ASD | TC |
| --- | --- | --- |
| GU | 26 | 21 |
| KKI | 39 | 108 |
| NYU | 80 | 55 |
| OHSU | 22 | 34 |
| SDSU | 13 | 12 |
| STANFORD | 13 | 14 |
| UCLA | 22 | 22 |
| UM | 10 | 16 |
|  | 225 | 282 |

**Table S2** | Brain regions with significantly altered rsFC in the hippocampal subregions of ASD patients compared to healthy controls.

| Right whole hippocampus | | Left caudal hippocampus | | Right rostral hippocampus | | Right caudal hippocampus | |
| --- | --- | --- | --- | --- | --- | --- | --- |
| Brain regions | Voxels | Brain regions | Voxels | Brain regions | Voxels | Brain regions | Voxels |
| Precentral_L | 77 | Frontal_Sup_2_L | 135 | Precentral_L | 88 | Rolandic_Oper_L | 23 |
| Frontal_Sup_2_L | 108 | Frontal_Mid_2_L | 173 | Frontal_Sup_2_L | 16 | Lingual_R | 34 |
| Rolandic_Oper_L | 20 | Calcarine_L | 82 | Olfactory_L | 10 | Fusiform_R | 10 |
| Supp_Motor_Area_L | 21 | Cuneus_L | 112 | Rectus_L | 14 | Parietal_Sup_L | 105 |
| OFCpost_L | 14 | Cuneus_R | 36 | OFCmed_L | 29 | Parietal_Inf_L | 188 |
| Insula_L | 69 | Lingual_L | 77 | OFCpost_L | 27 | SupraMarginal_L | 69 |
| Calcarine_L | 16 | Occipital_Sup_L | 192 | Insula_L | 95 | Cerebellum_Crus1_L | 192 |
| Calcarine_R | 21 | Occipital_Sup_R | 92 | Occipital_Sup_L | 48 | Cerebellum_Crus1_R | 26 |
| Cuneus_L | 61 | Occipital_Mid_L | 198 | Occipital_Mid_L | 132 | Cerebellum_Crus2_L | 115 |
| Lingual_L | 28 | Occipital_Mid_R | 53 | Postcentral_L | 41 | Cerebellum_6_L | 55 |
| Lingual_R | 42 | Fusiform_L | 77 | Parietal_Inf_L | 165 | Cerebellum_6_R | 50 |
| Occipital_Sup_L | 110 | Cerebellum_Crus1_L | 165 | SupraMarginal_L | 140 | Cerebellum_7b_L | 41 |
| Occipital_Mid_L | 120 | Cerebellum_Crus1_R | 85 | Caudate_R | 50 | Cerebellum_8_L | 26 |
| Postcentral_L | 102 | Cerebellum_Crus2_L | 27 | Putamen_L | 132 |  |  |
| Parietal_Sup_L | 201 | Cerebellum_4_5_L | 61 | Putamen_R | 15 |  |  |
| Parietal_Inf_L | 304 | Cerebellum_6_L | 158 | Pallidum_L | 21 |  |  |
| SupraMarginal_L | 155 | Cerebellum_6_R | 56 | Temporal_Sup_L | 11 |  |  |
| Precuneus_L | 19 | Cerebellum_8_L | 11 | Temporal_Mid_L | 111 |  |  |
| Putamen_L | 94 | Cerebellum_8_R | 27 | Temporal_Inf_L | 15 |  |  |
| Temporal_Sup_L | 16 |  |  | Thalamus_L | 140 |  |  |
| Temporal_Mid_L | 56 |  |  | Thalamus_R | 58 |  |  |
| Temporal_Inf_L | 15 |  |  |  |  |  |  |
| Thalamus_L | 19 |  |  |  |  |  |  |
| Cerebellum_Crus1_L | 220 |  |  |  |  |  |  |
| Cerebellum_Crus1_R | 75 |  |  |  |  |  |  |
| Cerebellum_Crus2_L | 146 |  |  |  |  |  |  |
| Cerebellum_6_L | 41 |  |  |  |  |  |  |
| Cerebellum_6_R | 58 |  |  |  |  |  |  |
| Cerebellum_7b_L | 42 |  |  |  |  |  |  |
| Cerebellum_8_L | 25 |  |  |  |  |  |  |

Note: Brain regions with voxel counts greater than 10 were shown based on AAL3 atlas.

**Table S3** | Linear SVM and Logistic Regression classifiers results based on significant group differences in hippocampal subregion rsFC.

|  | Region | Accuracy | Sensitivity | Specificity | Precision | F1-score | AUC |
| --- | --- | --- | --- | --- | --- | --- | --- |
| Linear SVM | Left caudal | 0.381 | 0.433 | 0.316 | 0.442 | 0.437 | 0.659 |
|  | Right rostral | 0.387 | 0.468 | 0.284 | 0.451 | 0.459 | 0.669 |
|  | Right caudal | 0.406 | 0.489 | 0.302 | 0.468 | 0.478 | 0.638 |
|  | Fused | 0.341 | 0.408 | 0.258 | 0.408 | 0.408 | 0.735 |
| Logistic Regression | Left caudal | 0.367 | 0.418 | 0.302 | 0.429 | 0.424 | 0.325 |
|  | Right rostral | 0.371 | 0.443 | 0.280 | 0.436 | 0.439 | 0.318 |
|  | Right caudal | 0.394 | 0.479 | 0.289 | 0.458 | 0.468 | 0.335 |
|  | Fused | 0.353 | 0.415 | 0.276 | 0.418 | 0.416 | 0.270 |

Fused: Significant differences from the three hippocampal subregions were combined for classifier training. These results represent the model evaluation with five-fold cross-validation.

**Table S4** | Significant associations between subscales of the SRS and RBS-R questionnaires and rsFC of different hippocampal subregions.

| SRS: AWARENESS  Right caudal hippocampus | | COMPULSIVE  Right rostral hippocampus | | RESTRICTED  Right caudal hippocampus | | SELF-INJURIOUS  Right caudal hippocampus | |
| --- | --- | --- | --- | --- | --- | --- | --- |
| Brain regions | Voxels | Brain regions | Voxels | Brain regions | Voxels | Brain regions | Voxels |
| Fusiform_R | 24 | Fusiform_L | 25 | Frontal_Sup_2_L | 149 | Precentral_R | 29 |
| Temporal_Mid_R | 20 | Cerebellum_Crus1_L | 22 | Frontal_Mid_2_L | 38 | Supp_Motor_Area_R | 23 |
| Temporal_Inf_R | 44 | Cerebellum_4_5_L | 14 | Frontal_Sup_Medial_L | 16 | Postcentral_R | 113 |
|  |  | Cerebellum_6_L | 28 |  |  | Parietal_Sup_R | 24 |
|  |  | Cerebellum_7b_L | 32 |  |  | Parietal_Inf_R | 141 |
|  |  | Cerebellum_8_L | 104 |  |  | SupraMarginal_R | 94 |
|  |  | Cerebellum_9_L | 24 |  |  | Angular_R | 37 |
|  |  |  |  |  |  | Precuneus_R | 13 |
|  |  |  |  |  |  | Paracentral_Lobule_R | 31 |

Note: Brain regions with voxel counts greater than 10 were shown based on AAL3 atlas.

**Table S5** | Significant associations between subscales of the age subscales of the ADI-R questionnaire and rsFC in different hippocampal subregions.

| Left rostral hippocampus | | Right rostral hippocampus | | Left caudal hippocampus | | Right caudal hippocampus | |
| --- | --- | --- | --- | --- | --- | --- | --- |
| Brain regions | Voxels | Brain regions | Voxels | Brain regions | Voxels | Brain regions | Voxels |
| Precentral_L | 126 | Precentral_L | 159 | Frontal_Inf_Oper_R | 17 | Precentral_R | 100 |
| Frontal_Sup_2_L | 100 | Precentral_R | 102 | Frontal_Inf_Orb_2_R | 10 | Frontal_Sup_2_L | 15 |
| Frontal_Sup_2_R | 235 | Frontal_Sup_2_L | 135 | Supp_Motor_Area_L | 49 | Frontal_Sup_2_R | 29 |
| Frontal_Mid_2_L | 14 | Frontal_Sup_2_R | 194 | Supp_Motor_Area_R | 179 | Frontal_Inf_Oper_R | 20 |
| Frontal_Mid_2_R | 126 | Frontal_Mid_2_L | 165 | Olfactory_R | 11 | Frontal_Inf_Orb_2_L | 22 |
| Frontal_Inf_Oper_L | 14 | Frontal_Mid_2_R | 122 | Rectus_R | 11 | Frontal_Inf_Orb_2_R | 21 |
| Frontal_Inf_Oper_R | 19 | Frontal_Inf_Oper_L | 48 | OFCant_R | 17 | Supp_Motor_Area_L | 280 |
| Frontal_Inf_Orb_2_L | 15 | Frontal_Inf_Oper_R | 16 | OFCpost_R | 29 | Supp_Motor_Area_R | 240 |
| Frontal_Inf_Orb_2_R | 13 | Frontal_Inf_Tri_L | 42 | Insula_R | 79 | Frontal_Sup_Medial_L | 11 |
| Supp_Motor_Area_L | 140 | Frontal_Inf_Orb_2_L | 16 | Cingulate_Mid_L | 33 | OFCpost_R | 27 |
| Supp_Motor_Area_R | 264 | Frontal_Inf_Orb_2_R | 15 | Cingulate_Mid_R | 79 | Insula_L | 109 |
| Olfactory_R | 27 | Rolandic_Oper_R | 27 | Putamen_R | 28 | Insula_R | 109 |
| Frontal_Sup_Medial_L | 20 | Supp_Motor_Area_L | 286 | ACC_sup_L | 51 | Cingulate_Mid_L | 112 |
| Frontal_Sup_Medial_R | 24 | Supp_Motor_Area_R | 266 | ACC_sup_R | 27 | Cingulate_Mid_R | 83 |
| Rectus_R | 12 | Olfactory_R | 20 |  |  | Calcarine_L | 73 |
| OFCmed_R | 14 | Frontal_Sup_Medial_L | 29 |  |  | Calcarine_R | 59 |
| OFCant_L | 14 | OFCpost_L | 41 |  |  | Cuneus_L | 33 |
| OFCant_R | 15 | OFCpost_R | 41 |  |  | Cuneus_R | 102 |
| OFCpost_L | 56 | Insula_L | 62 |  |  | Occipital_Sup_L | 27 |
| OFCpost_R | 67 | Insula_R | 123 |  |  | Occipital_Sup_R | 55 |
| Insula_L | 68 | Cingulate_Mid_L | 96 |  |  | Occipital_Mid_R | 26 |
| Insula_R | 64 | Cingulate_Mid_R | 111 |  |  | Postcentral_R | 71 |
| Cingulate_Mid_L | 104 | Amygdala_L | 21 |  |  | SupraMarginal_L | 20 |
| Cingulate_Mid_R | 126 | Calcarine_L | 51 |  |  | Paracentral_Lobule_R | 14 |
| Amygdala_R | 11 | Calcarine_R | 20 |  |  | Putamen_L | 95 |
| Postcentral_L | 20 | Cuneus_L | 93 |  |  | Temporal_Sup_L | 84 |
| Parietal_Inf_R | 31 | Cuneus_R | 72 |  |  | Temporal_Sup_R | 12 |
| SupraMarginal_R | 163 | Occipital_Sup_R | 46 |  |  | Temporal_Pole_Sup_R | 48 |
| Angular_R | 13 | Occipital_Mid_R | 67 |  |  | Temporal_Mid_L | 88 |
| Putamen_L | 40 | Postcentral_L | 21 |  |  | ACC_sup_L | 91 |
| Putamen_R | 10 | SupraMarginal_L | 120 |  |  | ACC_sup_R | 33 |
| Temporal_Sup_R | 68 | SupraMarginal_R | 171 |  |  |  |  |
| Temporal_Pole_Sup_L | 17 | Putamen_L | 29 |  |  |  |  |
| Temporal_Pole_Sup_R | 43 | Putamen_R | 14 |  |  |  |  |
| ACC_sup_L | 127 | Pallidum_L | 14 |  |  |  |  |
| ACC_sup_R | 87 | Temporal_Sup_L | 59 |  |  |  |  |
|  |  | Temporal_Sup_R | 52 |  |  |  |  |
|  |  | Temporal_Pole_Sup_L | 26 |  |  |  |  |
|  |  | Temporal_Pole_Sup_R | 59 |  |  |  |  |
|  |  | Temporal_Mid_L | 18 |  |  |  |  |
|  |  | Temporal_Mid_R | 22 |  |  |  |  |
|  |  | ACC_sup_L | 101 |  |  |  |  |
|  |  | ACC_sup_R | 47 |  |  |  |  |
|  |  | Thalamus_L | 14 |  |  |  |  |

Note: Brain regions with voxel counts greater than 10 were shown based on AAL3 atlas.

**Table S6** | Random Forest classifier results based on whole-brain rsFC of different hippocampal subregions.

| Region | Accuracy | Sensitivity | Specificity | Precision | F1-score | AUC |
| --- | --- | --- | --- | --- | --- | --- |
| Left caudal | 0.531 | 0.723 | 0.289 | 0.560 | 0.632 | 0.555 |
| Right rostral | 0.548 | 0.741 | 0.307 | 0.573 | 0.646 | 0.552 |
| Right caudal | 0.554 | 0.752 | 0.307 | 0.576 | 0.652 | 0.514 |
| Fused | 0.560 | 0.766 | 0.302 | 0.579 | 0.660 | 0.550 |

Fused: Significant differences from the three hippocampal subregions were combined for classifier training. These results represent the model evaluation with five-fold cross-validation.

**Whole-brain analysis**

We used 48 anatomical regions of interest (ROIs) defined by the Human Brainnetome Atlas (www.brainnetome.org) and extracted the mean time series from each ROI. We calculated the resting-state functional connectivity (FC) strength (Pearson correlation) between each ROI-pair as FC, forming a 48 ×48 FC matrix. We then Z-transformed all FCs with a Fisher’s r-to-z transformation and applied ComBat harmonization to remove site effects. Subsequently, we applied a general linear model (GLM) to perform independent regression analyses for each functional connectivity (FC) feature. Specifically, the strength of each FC was treated as the dependent variable, with group (ASD vs. TC) as the primary independent variable, while age, IQ, and head motion were included as covariates to control for potential confounding effects. For each FC, we extracted the t-statistic and uncorrected p-value for the group effect from the model to quantify the effect size and statistical significance of functional connectivity differences between the ASD and TC groups. Finally, to address the issue of multiple comparisons, p-values obtained from all FC tests were corrected using the false discovery rate (FDR) method (q < 0.05).

We chose this atlas for analysis because the subsequent segmentation of hippocampal subregions was also based on it. However, this atlas does not include the cerebellum. Therefore, we additionally conducted analyses using the AAL template (116 anatomical regions), which contains cerebellar regions. The analysis procedures were consistent with those described above.

Group-level whole-brain analyses based on both the Human Brainnetome atlas (Figure S1) and the AAL atlas (Figure S2) revealed a series of potential connectivity alterations; however, none of these FCs survived stringent whole-brain multiple-comparison correction. Both analyses revealed significant differences in hippocampal functional connectivity with multiple brain regions between the ASD and control groups when multiple comparison correction was not applied. These preliminary results provide important clues for further investigation of the hippocampus’s specific functional contributions in ASD, while also highlighting the necessity of validating these findings under more stringent statistical correction.


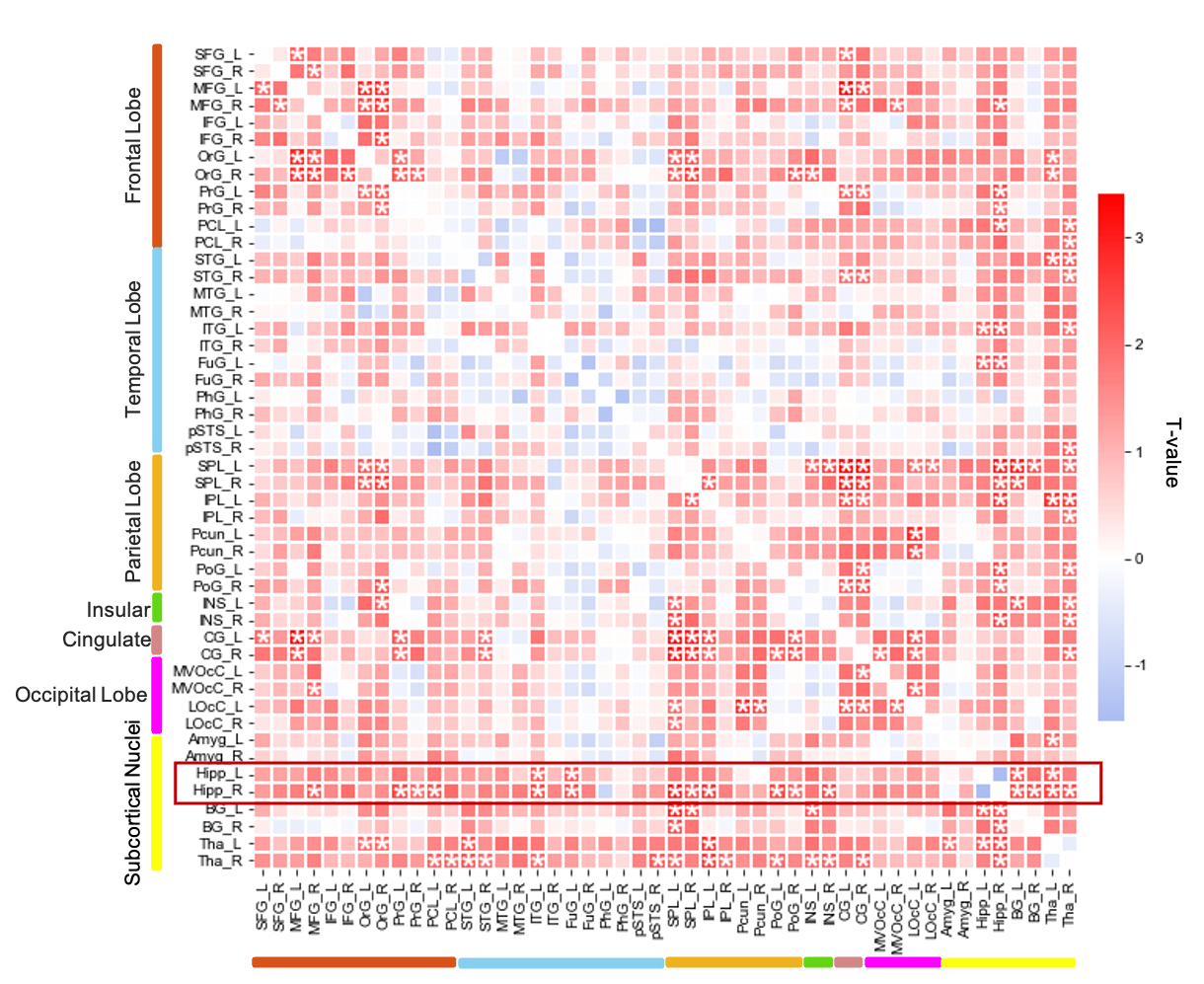


**Figure S1** | Group comparison of brain network connectivity analysis based on the Human Brainnetome Atlas. GLM was performed while controlling for age, IQ, and head motion as covariates. The above results are uncorrected for FDR. *: Indicates the significant result. Red connections represent enhanced FC in ASD compared to TC, while blue connections represent decreased FC. The red boxes indicate the left and right hippocampus.


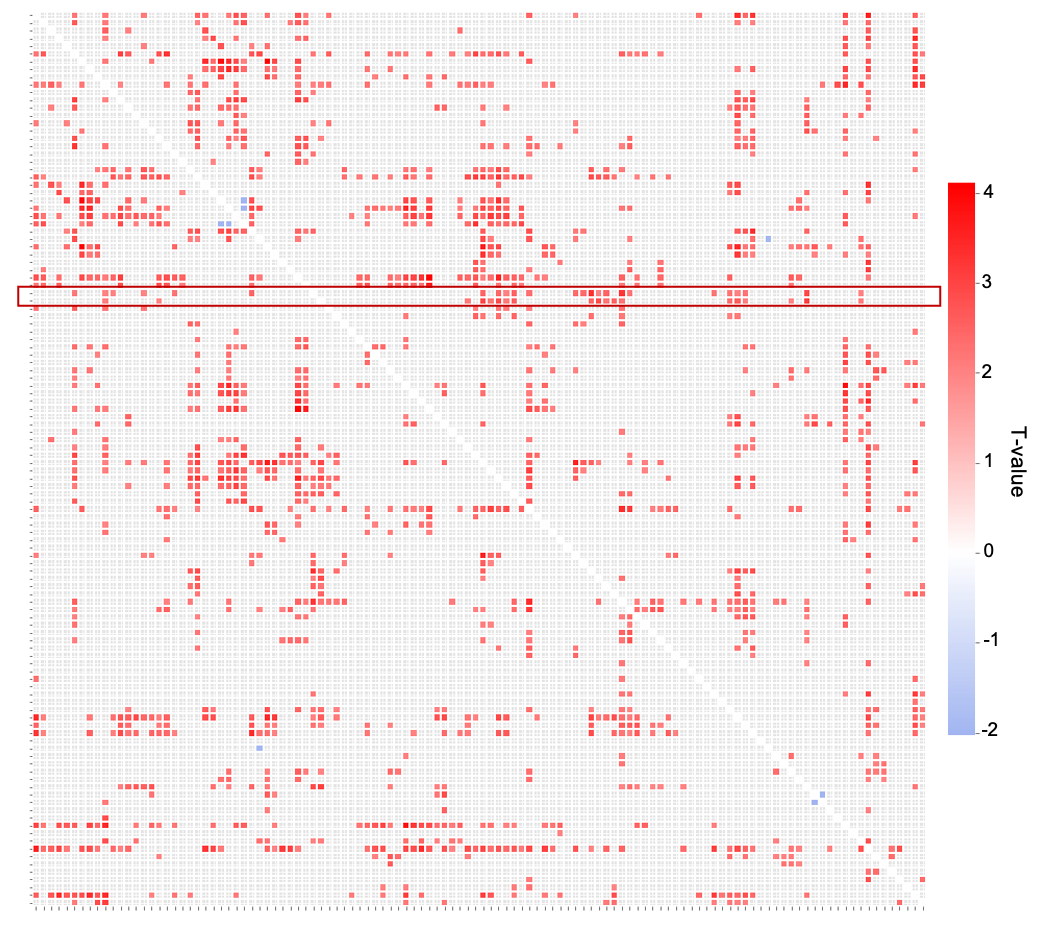


**Figure S2** | Group comparison of brain network connectivity analysis based on the AAL atlas. The above results are uncorrected for FDR. Red connections represent enhanced FC in ASD compared to TC, while blue connections represent decreased FC. The y-axis is arranged from top to bottom, and the x-axis from left to right, corresponding to the 116 regions of the AAL atlas, labeled 1 to 116. The red boxes indicate the left and right hippocampus.
